# Apical Dimension of Root Canal Clinically Assessed with and without Periapical Lesions

**DOI:** 10.1155/2014/374971

**Published:** 2014-06-24

**Authors:** Andrea Gesi, Paolo Mareschi, Tiziana Doldo, Marco Ferrari

**Affiliations:** ^1^School of Dentistry, University of Siena, Ospedale Le Scotte, Viale Mario Bracci 16, 53100 Siena, Italy; ^2^Dental Material and Prosthodontics, University of Siena, 53100 Siena, Italy

## Abstract

To clinically evaluate the dimension of the more apical extent of the root canal after appropriate preflaring in the case of primary treatment and retreatment with and without the presence of periapical radiolucency, 392 single-rooted teeth with only one canal were evaluated during endodontic therapy. The canals were divided in two groups depending on the presence or absence of periapical radiolucency. After preflaring of the root canal the size of the root canal terminus (apical canal dimension) was gauged with hand-held Light Speed LS1 files inserted at the estimated working length and established with the use of an electronic apex locator. The dimension recorded in the computer database was represented by the largest instrument able to reach the electronically established working length. The differences between the treatment groups were assessed using the Mann-Whitney *U* test and the significance level was set at *P* < 0.05. Teeth with lesions had a significantly greater diameter in the apical region than teeth without lesions (*P* < 0.001). The dimension of the apical portion of the root canal is larger in the case of periapical radiolucency. This involves verifying this parameter in order to use the correct sized instruments and to obtain an efficient cutting action at the apical level.

## 1. Introduction

The significance of cleaning and shaping root canals properly for successful endodontic therapy is well established. Three parameters are considered critical: the length of the canal preparation in relation to the root apex, the so-called working length, its taper, and the horizontal dimension of its most apical extent [1–7]. To achieve the desirable shape it is essential that the cross-sectional diameter of the finished canal preparation gradually decreases towards the root canal terminus [2, 3]. This geometrical shape is necessary to ensure a correct action of the endodontic instruments, obtain the most effective cutting action against the canal walls and, at the same time, determine the conditions for a correct fit of the tapered master cone of gutta-percha. By balancing the shaping of the canal to its anatomy we can instrument the root canal, reducing the risk of overpreparation, especially in the apical region, and create the conditions for an accurate mechanical cleaning and aid the action of the irrigant solution [8–11]. In order to guide the instrumentation to give an adequate shape, it has been considered important to establish the apical diameter with the aid of a root canal instrument [12–16]. However, some authors question the potential of getting an accurate recording and sustain the fact that this measurement is generally lower with respect to the true dimension of the root end portion [4, 5]. Especially in oval-shaped apical foramina the gauging may record only the minor diameter [4–6]. A complicating factor, not invariably recognized, is that in cases with apical periodontitis the apical portion of the root may have been resorbed to an extent that the apical canal dimension is enlarged. Thus, these factors may reduce the potential to achieve effective shaping and cleaning for optimal control of root canal infection. Nevertheless, studies emphasize the need to instrument large sizes in order to attain this objective: increasing the size of the apical instrumentation significantly reduces the number of remaining bacteria [8, 12, 17–25].

The aim of our study is to investigate the canal diameter in the apical region of tooth roots. The assessments were carried out in conjunction with routine endodontic treatments by the use of passive insertion of root canal instruments to the root canal terminus. Differences between teeth with and without periapical radiolucency were specifically evaluated. The null hypothesis that there is no difference in diameter between roots with and without periapical radiolucency was tested.

## 2. Materials and Methods 

### 2.1. Study Material

The patients making up the study material for this report were from private dental practices in Spilimbergo and Pisa, Italy. All patients were treated by two operators (P.M. and A.G.) between January 1, 2006, and December 31, 2009. The number of treated teeth was 428 in the same number of patients. The total number of teeth was single rooted with only one canal. These teeth were observed in the present study. To be considered for inclusion in the study the canals had to have complete root development and fully formed apices. The foramina also had to be patent and it had to be possible to instrument with the Light Speed file to the apex locator reading. The teeth with vital pulp had to respond positively to the pulp test while the necrotic teeth had to respond negatively to the pulp test and show periapical radiolucency on the preoperatory radiograph. A total of 36 teeth did not fulfil these criteria, leaving 392 teeth/root canals for the study. The teeth were divided in two groups depending on preoperative conditions. Group A included primary root canal treatments of teeth with vital pulps and without periapical radiolucency and group B consisted of teeth with nonvital pulp and with periapical lesions of endodontic origin. The presence or absence of periapical radiolucency was evaluated on the preoperative intraoral radiographs (Kodak DF58, Eastman Kodak Company, Rochester, NY) through observation by two endodontists, different to the operators. In the case of disagreement between the evaluators the worst assessment was considered and the tooth was discarded.

### 2.2. Clinical Procedure

Endodontic procedures were carried out according to well-proven clinical protocol. Briefly, all teeth were instrumented under rubber dam along a standardized access cavity following removal of caries and nonsustained restoration. If necessary the lost walls of the crown were rebuilt in order to maintain a correct reference point for the stopping point of the instruments.

A solution of 5.25% sodium hypochlorite (Niclor, Ogna, Italy) was used for irrigation.

In both groups the instrumentation process was initiated with #10 and #15 K-files (Maillefer, Switzerland) to eliminate possible interferences in the coronal and medium third of the canal with the intent not to interfere with the apical third. A #08 K-file was then applied to check apical patency.

The working length was established with an electronic apex locator (Root ZX, Morita, Japan) connected with the #08 K-file or larger if appropriate. A preflaring of the canal was conducted with a #10, #15, and #20 Mtwo NiTi rotary file (Sweden & Martina, Due Carrare, Padua, Italy) running 2 mm short of the working length.

The size of the apical terminus was gauged with hand-held Light Speed LS1 files (Light Speed, San Antonio, USA) according to the following procedure. First, a thin instrument, size of #20 or larger if necessary, was inserted in the canal at the estimated working length. Successively larger files were tried at the same level until an instrument could not be passed beyond that depth. Once connected to the electronic apex locator the correct position was subsequently determined. The apical fit was then evaluated again. In all instances a larger Light Speed file was subsequently tried to ensure that it could not be taken to the same depth. The file size representing the minor diameter was considered the largest one capable of arriving at the working length. After the measuring procedure described above, the endodontic treatment was completed.

The final file size obtained at working length was entered into a computer database and analyzed. The Mann-Whitney *U* test was applied to assess the statistical significance of the differences in diameter between teeth with and without lesions. The level of significance was set at *P* < 0.05.

## 3. Results

A total of 428 teeth, single rooted and with only one canal, were considered for the present study. 36 teeth were eliminated, 16 in group A of vital teeth without periapical lesions and 18 in group B of necrotic teeth with periapical radiolucency. In particular, in group A it was not possible to negotiate the apical foramen in 15 canals and in 1 case it was not possible to bring the Light Speed instrument to the terminus of the canal because of a severe apical curvature. In group B 13 canals were blocked in the apical region and it was not possible to use the Light Speed file up to the apical foramen in only one case. In group B 3 more teeth were discarded because, although they had nonvital pulp, periapical radiolucency was not shown in the preoperative radiograph. 392 teeth fulfilled the criteria and were included and evaluated in the study.

**Figure 1:**
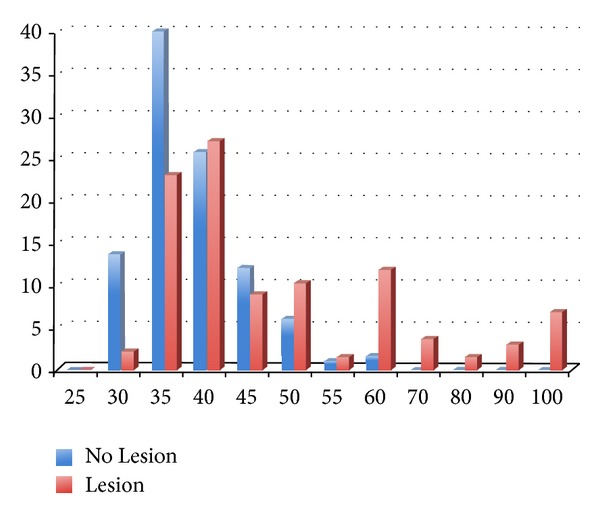
Data comparison of the 2 groups.

The Mann-Whitney *U* test revealed that teeth with lesions had a significantly greater diameter in the apical region than teeth without lesions (*P* < 0.001) (Table 1). In cases with periapical radiolucency, larger instruments can be taken to the working length than in cases without lesions (Figure 1).

## 4. Discussion

In accordance with the results of this clinical study the null hypothesis was rejected. In fact roots without periapical translucency showed a wider diameter at the apex than those without periapical translucency. This result can be explained by the frequent association between the presence of periapical inflammatory lesions and apical root resorption [26, 27].

Although there are different opinions about the width to which the apical portion of root canals should be prepared in endodontics, accurate measurement of the apical dimension should provide a better basis for the debridement of the root canal space especially in cases of infected root canals [28]. Determination by tactile sense which is often employed has been defined as empiric and unreliable by some authors [4, 29]. Studies conducted by the use of microcomputed tomography scans confirmed, for example, that the fit of the initial apical file was poor [5]. Moreover, in approximately one-fourth of the canals the apical portion has an oval-shaped section and the long canal diameter is equal to or larger than twice the short canal diameter and in this case the recorded diameter is the smaller of the two [4]. From these studies it can be assumed that clinical assessment of the apical diameter has a margin of error that cannot be avoided but reduced. Based on observation of the apical diameter in human teeth [22–24] some authors have in fact suggested that the apical portion of the root canal should be enlarged three sizes larger than the first file that clinically binds to the working length in order to obtain a good debridement of the apical region [30–32]. A recent clinical study concluded that in simple root canal systems only apical instrumentation larger than the recommended size might reduce the debris and number of remaining bacteria in this area of the canal [16, 21–25, 28–35]. They have also shown that larger apical size yields cleaner canals that may promote further success. Failing to clean canals, especially in the apical region, can result in treatment failure [34, 35].

Furthermore, being able to evaluate the minor diameter and knowing that a value inferior to that of the real dimension is often recorded in the clinic can lead to a better decision and strategy with regard to the appropriate final diameter needed for complete apical shaping and to obtain the correct shape, cleaning, and sealing of the whole root canal.

Preflaring the middle and coronal portion of the canal is recommended prior to determining the apical diameter [12, 33] and the use of nontapered instruments is suggested in order to easily bypass any interference in the root canal that might lead to premature binding [12, 21, 28]. In the present study the operators used all the possible clinical procedures and instruments indicated in the literature to clinically obtain the best results in determining the dimension of the apical portion of the root canal, using the apex locator to determine the working length. The aim of the study was not to determine the apical diameter, represented by the first file that binds at the working length, but the dimension of the apical region at the end of the canal, recording the largest file able to arrive at the working length. In fact, in many cases, after having found the first file that engages at the working length and cannot proceed further, it is possible to verify that another 2-3 larger files can also passively reach the same length. This event suggests that often the size of the first binding file at working length is not enough to create mechanical contact with the dentinal walls of the canal in the last 2-3 millimeters of the apical area, confirming the data of previous study [16]. In this study the size of the largest file that can arrive at the working length has been considered the dimension of the more apical portion of the canal.

## 5. Conclusions

The results of the present study showed apical dimensions of a greater size with respect to the size normally recommended in the literature for a correct shaping of the canal. We can also deduce that many instrument series available ARE incomplete in terms of their apical dimension and unable to ensure the capacity to perform a correct cutting action on the dentinal root canal walls at the apical level. In particular, the presence of a periradicular inflammatory reaction determines the presence of a larger diameter compared to cases without periapical lesions. In any case information about the dimension of the canal in its apical portion is required in order to avoid overinstrumentation, with all its consequences, and to obtain, on the other hand, a correspondence in terms of size between the instruments and the canal to create a correct cleaning and shaping.

## Figures and Tables

**Table 1:** Ratio of diameters in the apical part of root canal in teeth with and without periapical lesions: all presented as median and interquartile ranges.

Group	*N*	Median	Interquartile ranges 25%–75%	Significance
Teeth with lesion	135	40	36.25–60	A
Teeth without lesion	257	35	35–40	B
